# Gene-specific machine learning for pathogenicity prediction of rare *BRCA1* and *BRCA2* missense variants

**DOI:** 10.1038/s41598-023-37698-6

**Published:** 2023-06-28

**Authors:** Moonjong Kang, Seonhwa Kim, Da-Bin Lee, Changbum Hong, Kyu-Baek Hwang

**Affiliations:** 1 Research Center, Software Division, NGeneBio, Seoul, 08390 Korea; 2 Department of Computer Science and Engineering, Graduate School, Soongsil University, Seoul, 06978 Korea

**Keywords:** Bioinformatics, Cancer genetics, Computer science

## Abstract

Machine learning-based pathogenicity prediction helps interpret rare missense variants of *BRCA1* and *BRCA2*, which are associated with hereditary cancers. Recent studies have shown that classifiers trained using variants of a specific gene or a set of genes related to a particular disease perform better than those trained using all variants, due to their higher specificity, despite the smaller training dataset size. In this study, we further investigated the advantages of “gene-specific” machine learning compared to “disease-specific” machine learning. We used 1068 rare (gnomAD minor allele frequency (MAF) < 0.005) missense variants of 28 genes associated with hereditary cancers for our investigation. Popular machine learning classifiers were employed: regularized logistic regression, extreme gradient boosting, random forests, support vector machines, and deep neural networks. As features, we used MAFs from multiple populations, functional prediction and conservation scores, and positions of variants. The disease-specific training dataset included the gene-specific training dataset and was > 7 × larger. However, we observed that gene-specific training variants were sufficient to produce the optimal pathogenicity predictor if a suitable machine learning classifier was employed. Therefore, we recommend gene-specific over disease-specific machine learning as an efficient and effective method for predicting the pathogenicity of rare *BRCA1* and *BRCA2* missense variants.

## Introduction

*BRCA1* and *BRCA2* (*BRCA1*/*2*) genes are associated with an elevated risk of developing breast and ovarian cancers^1,2^. Small germline variants of *BRCA1/2* are one of the primary sources of such risk^3–5^. Meanwhile, next-generation sequencing technologies are rapidly being integrated into clinical practice, identifying vast amounts of small germline *BRCA1/2* variants^6–8^. Accurate interpretation of the identified variants is one of the critical elements of clinical practice. Unlike synonymous and common missense variants, which are usually benign, and nonsense and frameshift variants, which are often pathogenic^9^, rare missense variants of *BRCA1/2* are hard to interpret^10^. In this regard, computational prediction of the pathogenicity of rare *BRCA1/2* missense variants can help the interpretation process^11,12^.

Supervised machine learning has been widely adopted to develop computational tools for the pathogenicity prediction of variants, including rare missense ones^13–25^. A prediction tool based on supervised machine learning takes a set of features, such as minor allele frequencies (MAFs), predicted functional impacts of a variant, and the degree of conservation across multiple species at its genomic position, as input. A training dataset containing known pathogenic and benign variants is used to build a pathogenicity predictor in supervised machine learning. According to the composition of training variants, supervised machine learning for variant pathogenicity prediction is divided into genome-wide, disease-specific, and gene-specific.

Genome-wide supervised machine learning approaches use variants from across the whole genome to develop pathogenicity predictors. Popular examples include REVEL^19^, BayesDel^17^, and ClinPred^13^. One advantage of the genome-wide approach is that it involves a larger number of training variants, which can improve the performance of the learned model by reducing variance^26^. However, this approach does not account for disease-specific patterns in variant pathogenicity. For example, the pathogenicity of a variant could be different between a hereditary cancer syndrome and a hereditary cardiovascular disease. Disease-specific supervised machine learning addresses this issue by using only disease-specific variants, i.e., variants of a set of genes related to a specific disease or a group of similar disorders. Evans et al. developed pathogenicity predictors specific to each of cardiomyopathy, epilepsy, and RASopathies using disease-specific supervised machine learning^16^. These disease-specific predictors were found to outperform genome-wide pathogenicity predictors. Lai et al. showed that hereditary cancer-specific and cardiovascular disorder-specific predictors worked better than genome-wide predictors^20^. Zhang et al. also showed that the disease-specific approach is better than the genome-wide method for inherited cardiomyopathies and arrhythmias^21^.

Compared to the disease-specific approach, gene-specific supervised machine learning is even more specific as it builds pathogenicity predictors using variants from only a particular disease gene, e.g., *BRCA1* or *BRCA2*. This method has the potential to perform best due to its highest specificity; however, its training variants are most limited. In this sense, it is likely to perform poorly due to high variance. Crockett et al.^22^, Padilla et al.^25^, Hart et al.^18^, Aljarf et al.^14^, Khandakji and Mifsud^24^, and Karalidou et al.^23^ have developed gene-specific variant pathogenicity predictors for disease-associated genes, including *BRCA1* and *BRCA2*. Most of these studies showed that gene-specific predictors performed better than or comparable to genome-wide predictors. However, none of them have compared their gene-specific approach with the disease-specific approach, which is less specific but expected to have less variance.

In this study, we investigated the efficacy of gene-specific supervised machine learning in predicting the pathogenicity of rare *BRCA1*/*2* missense variants, compared to the disease-specific approach. Our work differs from the previous studies that focused on gene-specific machine learning to predict the pathogenicity of variants. First, they did not compare the gene-specific and disease-specific approaches. They compared the gene-specific approach with the genome-wide approach^14,18,22,24,25^ or did not make any comparison^23^. The comparison between gene-specific and disease-specific approaches is meaningful because there is a trade-off between specificity and training sample size. In addition, the previous works focused only on *BRCA2*^24^, used a single machine learning algorithm^24^, or did not optimize the hyperparameters of the machine learning algorithm^14,24,25^. Furthermore, none of the previous works, except one study^18^, used the performance measure known to be more informative than others in imbalanced classification: the area under the precision-recall curve (AUPRC)^27–29^. For the investigation, we used rare missense variants of 28 genes associated with hereditary cancers, including *BRCA1/2*. We employed a set of widely used linear and non-linear machine learning methods: the lasso, ridge, elastic net, extreme gradient boosting (XGBoost), random forests (RFs), support vector machines (SVMs), and deep neural networks (DNNs) to build the pathogenicity predictor. We evaluated and compared the performance of each machine learning classifier when combined with either the gene-specific or disease-specific approach. These comparisons will provide insight into which of the two methods in which trade-off exists is better suited for the variant pathogenicity prediction.

## Methods

### Variant annotation and filtering

We downloaded a variant file in GRCh37 (clinvar_20200817.vcf.gz) from the ClinVar^30^ website (https://www.ncbi.nlm.nih.gov/clinvar/). The downloaded VCF file was normalized using vt (version 0.5772)^31^ and in-house scripts. Then the normalized VCF file was annotated using SnpEff (version 4.3 s (build 2017-10-25 10:05))^32^, SnpSift (version 4.3 s (build 2017-10-25 10:05))^33^, and Ensembl Variant Effect Predictor (VEP) (version 86)^34^. The databases used for annotation were dbSNP (build 151)^35^, dbscSNV (version 1.1)^36^, gnomAD (release 2.1.1)^37^, Korean Variant Archive (KOVA)^38^, Korean Reference Genome Database (KRGDB) (phase 2)^39^, and dbNSFP (version 4.1a)^40^. In total, 769,966 variants were annotated. The annotated variants were filtered as follows. First, only the variants of which clinical significance in ClinVar is Benign, Benign/Likely_benign, Likely_benign, Pathogenic, Pathogenic/Likely_pathogenic, or Likely_pathogenic were retained. Then, variants were filtered by the ClinVar review status. Only the variants of which review status is practice_guideline, reviewed_by_expert_panel, or criteria_provided_multiple_submitters,_no_conflicts were included in the experiments. Then, variants were filtered by type (single_nucleotide_variant in ClinVar’s annotation), MAF (gnomAD all populations < 0.005), and consequence (VEP consequence is missense_variant or missense_variant&splice_region_variant). Finally, only the variants of 31 reportable transcripts of 30 genes associated with hereditary cancer syndromes compiled by Barrett et al.^41^ were used. Consequently, we used 1068 rare missense variants of 28 genes, including *BRCA1* and *BRCA2*. Among the 1068 variants, the numbers of *BRCA1* and *BRCA2* variants were 225 and 179, respectively. Supplementary Table S1 shows the number of variants per gene.

### Test variant sets

After the filtering, we grouped variants into two categories of clinical significance: P/LP (including Pathogenic, Pathogenic/Likely_pathogenic, and Likely_pathogenic) and B/LB (including Benign, Benign/Likely_benign, and Likely_benign). We determined the ratio of P/LP to B/LB variants in a test variant set in line with previous studies on the pathogenicity prediction of rare *BRCA1/2* missense variants since the class distribution of test examples influences the performance of a machine learning classifier^42^. In previous studies, the ratio of P/LP to B/LB test variants was 0.08^11^, 0.20^20^, and 0.22^12^ for *BRCA1* and 0.03^11^, 0.07^20^, and 0.07^12^ for *BRCA2*. From the 225 *BRCA1* variants, 86 were randomly selected and constituted a test variant set. The 86 variants of the test set included 14 P/LP variants (i.e., P/LP to B/LB ratio 0.19). Among the 79 variants of a test set chosen from the 179 *BRCA2* variants, the number of P/LP variants was six, making P/LP to B/LB ratio 0.08. We created ten test variant sets for each of *BRCA1* and *BRCA2* by repeated random subsampling.

### Training variant sets

**Figure 1:**
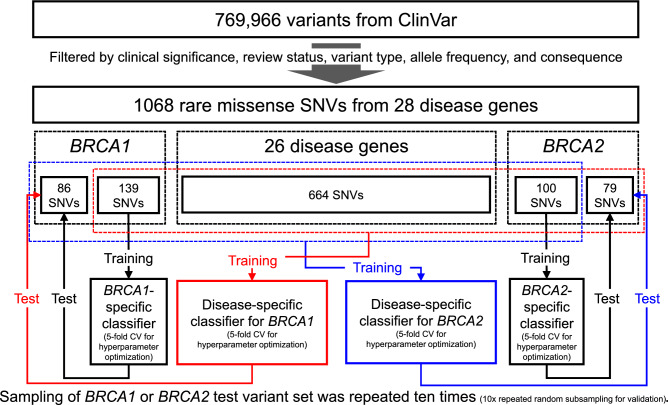
The workflow for constructing test and training variant sets for evaluating and comparing disease-specific and gene-specific machine learning.

We constructed gene-specific and disease-specific training variant sets for each of the ten test variant sets of *BRCA1* and *BRCA2*. For a test variant set of *BRCA1* (*BRCA2*) with 86 (79) variants, we used the remaining 139 *BRCA1* (100 *BRCA2*) variants as gene-specific training variants. The disease-specific training variants for a test variant set of *BRCA1* (*BRCA2*) consisted of the remaining 982 (989) variants from 28 genes, including *BRCA1* and *BRCA2*, associated with hereditary cancer syndromes. The entire workflow for constructing the test and training variant sets is shown in Fig. 1. Notably, the disease-specific training variant set for a *BRCA1* or *BRCA2* test variant set included the corresponding gene-specific training variant set.

### Features for variant pathogenicity prediction

We used five feature categories to predict the pathogenicity of rare *BRCA1/2* missense variants: MAF, site conservation score, predicted functional-impact score, position, and others. The features used in our study are listed in Supplementary Table S2. MAFs for 16 populations obtained from gnomAD, KOVA, KRGDB, and UK10K (from dbNSFP) were used as features. Missing values for the MAF features were replaced by zero. We created an additional feature indicating that MAF values were missing for each of the four MAF databases to discriminate between the missing and zero MAF values.

Furthermore, we used nine site conservation scores from dbNSFP as features. To minimize the risk of overfitting, we did not use functional impact scores, predicted by supervised machine learning models trained using variants labelled as pathogenic or benign, such as PolyPhen-2^43^ and Combined Annotation Dependent Depletion (CADD)^44^ scores. Consequently, only five predicted functional-impact scores from dbNSFP were used as features. LRT^45^, MutationAssessor^46^, SIFT^47^, and SIFT 4G^48^ had missing values among the five predicted functional-impact scores. Missing values of these four features were imputed by median over the training variant set. In addition, we created a missing status indicator for each of the LRT, MutationAssessor, and SIFT (including SIFT 4G) scores.

We also used four position features for a variant, i.e., the relative position of the exon in which it exists and its relative position in each of the cDNA, coding, and protein sequences. Finally, we used 12 “others” category features from gnomAD, dbNSFP, dbscSNV, and dbSNP. Among the 12 features, six had missing values. Two of them—dbNSFP_APPRIS and dbNSFP_codon_degeneracy—were categorical features, having “not annotated” as their values. Missing values of the four numerical features—gnomAD2_InbreedingCoeff, dbNSFP_LRT_Omega, dbscSNV_ADA_SCORE, and dbscSNV_RF_SCORE—were imputed by zero or median over the training variant set (see Supplementary Table S2 for details). Furthermore, we created two features respectively representing that the values of dbscSNV_ADA_SCORE and dbscSNV_RF_SCORE were missing. The two missing value indicators created for gnomAD MAF and LRT features were respectively used for indicating that gnomAD2_InbreedingCoeff and dbNSFP_LRT_Omega values were missing. In total, 55 features were used for variant pathogenicity prediction.

### Supervised machine learning methods

Before training, we centred and scaled each numeric or integer feature using its mean and standard deviation over the training variant set (the type of each feature is shown in Supplementary Table S2). We evaluated and compared eight supervised machine learning methods: three regularized logistic regression methods (the lasso, ridge, and elastic net), XGBoost, RFs, SVMs with the linear and radial basis function (RBF) kernels (Linear-SVMs and RBF-SVMs, respectively), and DNNs. We used the R caret package (version 6.0.-90) for training and testing the regularized logistic regression (method = ‘glmnet’), XGBoost (method = ‘xgbTree’), RF (method = ‘rf’), Linear-SVM (method = ‘svmLinear’), and RBF-SVM (method = ‘svmRadial’) models. We used the R keras package (version 2.9.0) for DNNs. We employed fully-connected feedforward DNNs with three hidden layers. The leaky rectified linear unit (ReLU) was used as an activation function for each node of the three hidden layers. We set the slope of leaky ReLU as 0.2. The activation function for the output layer was sigmoid. Since DNNs are known to perform worse on small datasets compared to other machine learning methods, we used the dropout technique to regularize them. The hyperparameter values of each method were optimized using five-fold cross-validation (CV) over the training variant set. The search range for each hyperparameter is shown in Tables 1 and 2. The AUPRC was used as the objective function for hyperparameter optimization. The AUPRC values were calculated using the R PRROC package (version 1.3.1).

**Table 1:** Optimized hyperparameters of the eight machine learning methods for gene-specific training. Hyperparameter names and search ranges are shown in R codes. XGBoost: extreme gradient boosting. RFs: random forests. Linear-SVMs: support vector machines with the linear kernel. RBF-SVMs: support vector machines with the radial basis function kernel. DNNs: deep neural networks.

Machine learning methods	Hyperparameters and search ranges
Lasso, ridge, and elastic net	lambda: c(seq(500, 200, by = − 100), seq(100, 10, by = − 10), 9:2, seq(1, 0.05, by = − 0.05), 0.01)
XGBoost	eta: c(0.05, 0.1, 0.15, 0.2) for *BRCA1*; c(0.05, 0.1, 0.15, 0.2, 0.25) for *BRCA2* nrounds: seq(50, 250, by = 10) for *BRCA1*; seq(50, 150, by = 10) for *BRCA2* gamma: c(0, 0.05) max_depth: 3:5 min_child_weight: 1:2
RFs	ntree: c(100, 300, 500, 1000, 3000) mtry: 1:15
Linear-SVMs	C: c(1, 10, 50)
RBF-SVMs	sigma: 2^seq(− 9, − 1, by = − 2) C: c(1, 10, 50)
DNNs	epochs: c(40, 80) lr: c(5e-5, 1e-5) dropout: c(0.4, 0.6) # dropout rate for hidden layers batch_size: c(40, 80) in_dropout: c(0.1, 0.2) # dropout rate for input layer hidden: c(1000, 3000) # number of units in a hidden layer

**Table 2:** Optimized hyperparameters of the eight machine learning methods for disease-specific training. Hyperparameter names and search ranges are shown in R codes. XGBoost: extreme gradient boosting. RFs: random forests. Linear-SVMs: support vector machines with the linear kernel. RBF-SVMs: support vector machines with the radial basis function kernel. DNNs: deep neural networks.

Machine learning methods	Hyperparameters and search ranges
Lasso, ridge, and elastic net	lambda: c(seq(100, 10, by = − 10), 9:2, seq(1, 0.05, by = − 0.05), 0.01)
XGBoost	eta: c(0.05, 0.1, 0.2, 0.3) nrounds: seq(100, 1000, by = 100) for *BRCA1*; seq(100, 500, by = 50) for *BRCA2* gamma: c(0, 0.05) max_depth: 3:5 min_child_weight: 1:3
RFs	ntree: c(100, 300, 500, 1000, 3000) mtry: 1:15
Linear-SVMs	C: c(1, 10, 50)
RBF-SVMs	sigma: 2^seq(− 9, − 1, by = − 2) C: c(1, 10, 50)
DNNs	epochs: c(40, 80) lr: c(5e-5, 1e-5) dropout: c(0.4, 0.6) # dropout rate for hidden layers batch_size: c(40, 80) in_dropout: c(0.1, 0.2) # dropout rate for input layer hidden: c(1000, 3000) # number of units in a hidden layer

## Results and discussion

### Prediction performance comparison of gene-specific and disease-specific machine learning

The ratio of pathogenic to benign *BRCA1*/*2* variants in the test variant set of our study is not balanced because it reflects the actual class distribution (see Methods). Therefore, we used AUPRC to evaluate the performance of pathogenicity predictors. The AUPRC is more informative for imbalanced classification datasets than other measures, such as accuracy and the area under the receiver operating characteristics curve (AUROC)^27–29^. Eight machine learning methods—ridge, lasso, elastic net, RFs, XGBoost, Linear- and RBF-SVMs, and DNNs—were employed for the performance comparison. Furthermore, we compared four popular genome-wide pathogenicity predictors: REVEL^19^, BayesDel^17^ with and without maximum allele frequency (MaxAF), and ClinPred^13^. In a recent study, REVEL and BayesDel performed better than other *in-silico* predictors^49^. ClinPred is a recently developed tool trained using ClinVar variants.

**Figure 2:**
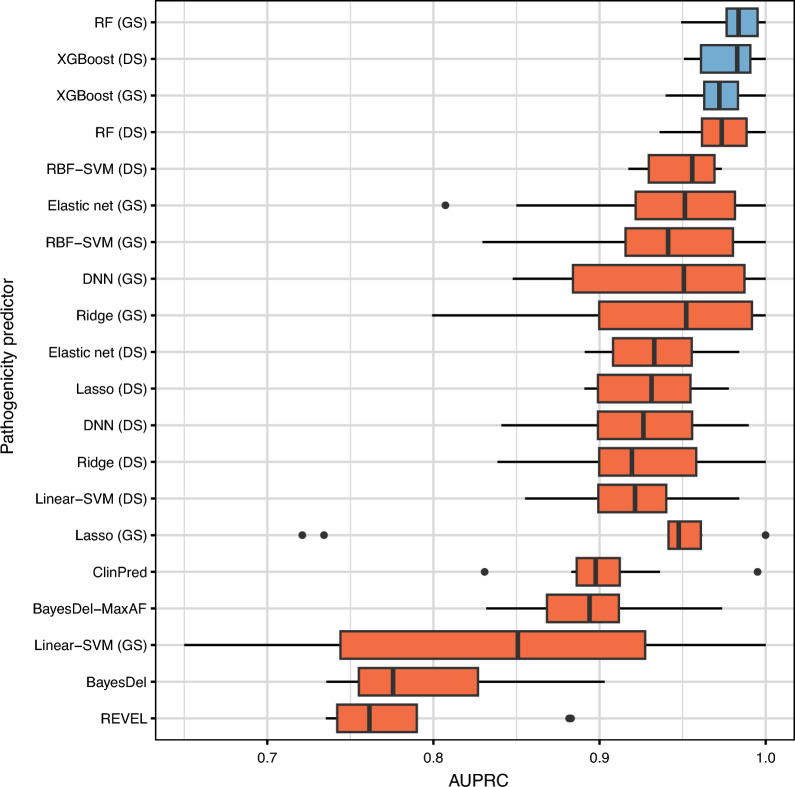
Prediction performance (in the area under the precision-recall curve (AUPRC)) of gene-specific (GS), disease-specific (DS), and genome-wide machine learning methods for rare *BRCA1* missense variants. All the methods are sorted by the average AUPRC on the ten test variant sets. The results of the best method and those not significantly outperformed by the best one (paired *t*-test *P* ≥ 0.05) are coloured in blue.

Figure 2 compares the performance of each method on *BRCA1*. We did not observe a remarkable difference in prediction performance between gene-specific and disease-specific machine learning. Disease-specific learning performed better than gene-specific learning when used with the lasso, XGBoost, Linear- and RBF-SVMs. For the other four methods, gene-specific learning was better than disease-specific learning. However, the performance difference between gene-specific and disease-specific learning was statistically significant (paired *t*-test *P* < 0.05) only for one machine learning model: RFs (see Supplementary Table S3). This result is noteworthy because the disease-specific training variant set was more than seven (= 982/139; see Methods) times larger than the gene-specific one. Moreover, the disease-specific training variant set includes all the gene-specific variants. It means that the variants from disease-associated genes other than *BRCA1* generally did not improve the pathogenicity prediction performance for *BRCA1*. Instead, the machine learning model substantially influenced pathogenicity prediction performance more than the training variant type. For *BRCA1*, the gene-specific RF achieved the highest AUPRC (0.9835 ± 0.0156). Two other models, i.e., XGBoost trained using the disease-specific and the gene-specific variant sets, were the second (AUPRC 0.9783 ± 0.0187) and the third (AUPRC 0.9727 ± 0.0176), respectively, showing comparable performance to the best method (paired *t*-test *P* = 0.1062 and 0.0801, respectively). All the others were statistically significantly worse than the gene-specific RF model (see Supplementary Table S4). The popular pathogenicity predictors trained using all genes, i.e., REVEL, BayesDel with and without MaxAF, and ClinPred, demonstrated poorer performance than the gene- and disease-specific machine learning approaches except for the Linear-SVM model, which performed worse than ClinPred and BayesDel with MaxAF.

**Figure 3:**
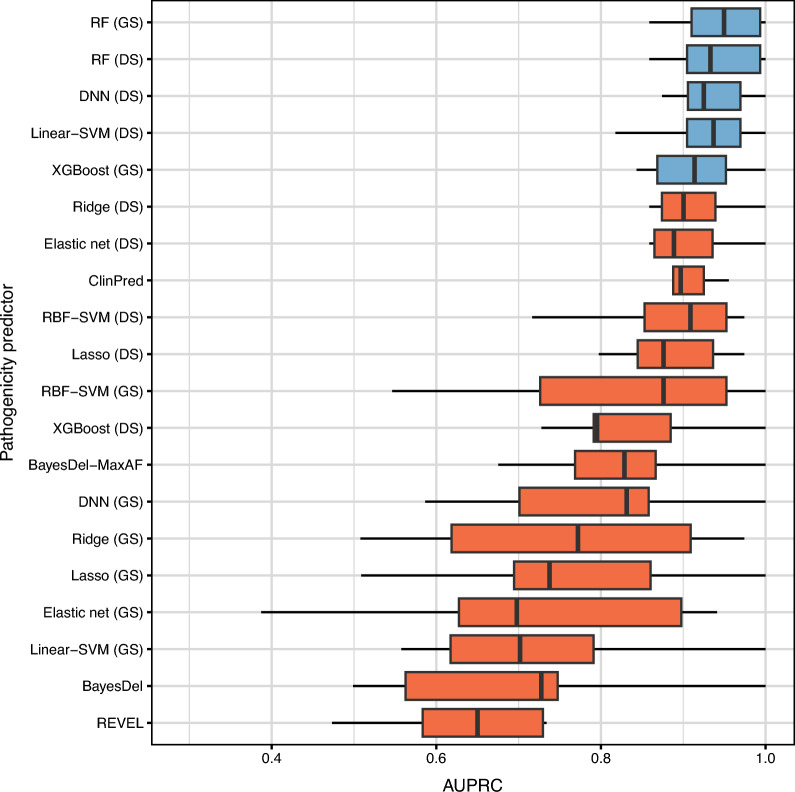
Prediction performance (in the area under the precision-recall curve (AUPRC)) of gene-specific (GS), disease-specific (DS), and genome-wide machine learning methods for rare *BRCA2* missense variants. All the methods are sorted by the average AUPRC on the ten test variant sets. The results of the best method and those not significantly outperformed by the best one (paired *t*-test *P* ≥ 0.05) are coloured in blue.

We show the comparison results for *BRCA2* in Fig. 3. Unlike the case of *BRCA1*, disease-specific learning generally performed better than gene-specific learning for *BRCA2*. Except for XGBoost and RFs, disease-specifically trained models showed higher AUPRC values than gene-specific ones. Moreover, the performance difference was statistically significant (paired *t*-test *P* < 0.05) for all machine learning models but RFs and RBF-SVMs (see Supplementary Table S5). However, gene-specific RFs achieved the best AUPRC (0.9467 ± 0.0483). Four other methods which obtained comparable performance (paired *t*-test *P* = 0.1436, 0.1693, 0.1575, 0.1035) to this were disease-specific RFs (AUPRC 0.9398 ± 0.0515), disease-specific DNNs (AUPRC 0.9331 ± 0.0413), disease-specific Linear-SVMs (AUPRC 0.9209 ± 0.0676), and gene-specific XGBoost (AUPRC 0.9167 ± 0.0581). All the other methods were statistically significantly worse than the gene-specific RF model (Supplementary Table S6). This result suggests that gene-specific learning is sufficient to obtain the optimal pathogenicity predictor for *BRCA2* if we use an appropriate machine learning algorithm. The popular pathogenicity predictors were not enough to attain high performances. Unlike the case of *BRCA1*, however, ClinPred and BayesDel with MaxAF showed higher AUPRC values than many gene-specific and disease-specific machine learning approaches (see Fig. 3).

We also compared the variance of ten trials between gene-specific and disease-specific machine learning. Because the gene-specific training variant set is much smaller than that of the disease-specific variant set (see Methods), the variance of gene-specific models is expected to be larger than that of disease-specific models. We show the comparison results for *BRCA1* and *BRCA2* in Supplementary Tables S3 and S5. For *BRCA1*, gene-specific learning showed statistically significantly higher variances (Pitman-Morgan test *P* < 0.05) than disease-specific learning for the lasso, elastic net, Linear- and RBF-SVMs among the eight machine learning models. However, the difference in variance was not statistically significant for the other four models, including RFs, which achieved the highest AUPRC. We observed a different result for *BRCA2*. The difference in variance was statistically significant (Pitman-Morgan test *P* < 0.05) for all but one machine learning method, meaning that *BRCA2*-specific training datasets were likely to produce more inconsistent results than much larger disease-specific training datasets. Interestingly, the variance of RFs, the best-performing predictor on *BRCA2*, was not statistically significantly different between gene-specific and disease-specific learning (Pitman-Morgan test *P* = 0.6321).

For reference, we also compared the gene-specific and disease-specific machine learning methods using the following performance measures: accuracy, sensitivity (i.e., recall), specificity, positive predictive value (PPV) (i.e., precision), F1 score, and AUROC. Comparative results for *BRCA1* and *BRCA2* are shown in Supplementary Tables 7 and 8, respectively. For *BRCA1*, both the gene-specific and disease-specific models were able to achieve optimal results for all of these performance measures when an appropriate machine learning algorithm was used. For accuracy, sensitivity, specificity, PPV, and F1 score, the gene-specific RF and the gene-specific and disease-specific XGBoost models performed optimally. For AUROC, the gene-specific and disease-specific DNN models performed best. For *BRCA2*, both the gene-specific and disease-specific methods achieved optimal results for accuracy, sensitivity, specificity, PPV, and F1 score when combined with an appropriate machine learning algorithm. However, for AUROC, only the disease-specific DNN model achieved optimal results. Interestingly, the DNN models outperformed the others in terms of AUROC for both *BRCA1* (gene- and disease-specific) and *BRCA2* (disease-specific only) genes, suggesting that DNNs may be more suitable than other machine learning models for obtaining optimal AUROC values. However, AUROC is known to be less informative and even misleading when evaluating the performance of a classifier on imbalanced datasets due to its misinterpretation of specificity^27^ and overly optimistic view^28,29^. The above results suggest that gene-specific variants are sufficient to obtain the optimal pathogenicity predictor for rare *BRCA1* and *BRCA2* missense variants when an appropriate machine learning algorithm is employed.

### Comparison of important features identified by gene-specific and disease-specific machine learning

As demonstrated in the preceding subsection, the selection of machine learning algorithms plays a more significant role than the type of training variants in achieving optimal pathogenicity predictions for rare *BRCA1/2* missense variants. Specifically, among the three predictors exhibiting optimal performance for *BRCA1*, two were trained on gene-specific variants, while one was trained on disease-specific variants. For *BRCA2*, two of the top five performing predictors employed gene-specific training variants (see Figs. 2 and 3). It is noteworthy that the top-performing model group for *BRCA1* comprised both gene-specific and disease-specific XGBoost models. As for *BRCA2*, the RF algorithm demonstrated the best performance regardless of the type of training variants used. We compared the significant features identified by these top-performing models obtained using the same machine learning algorithm but different types of training variants.

**Figure 4:**
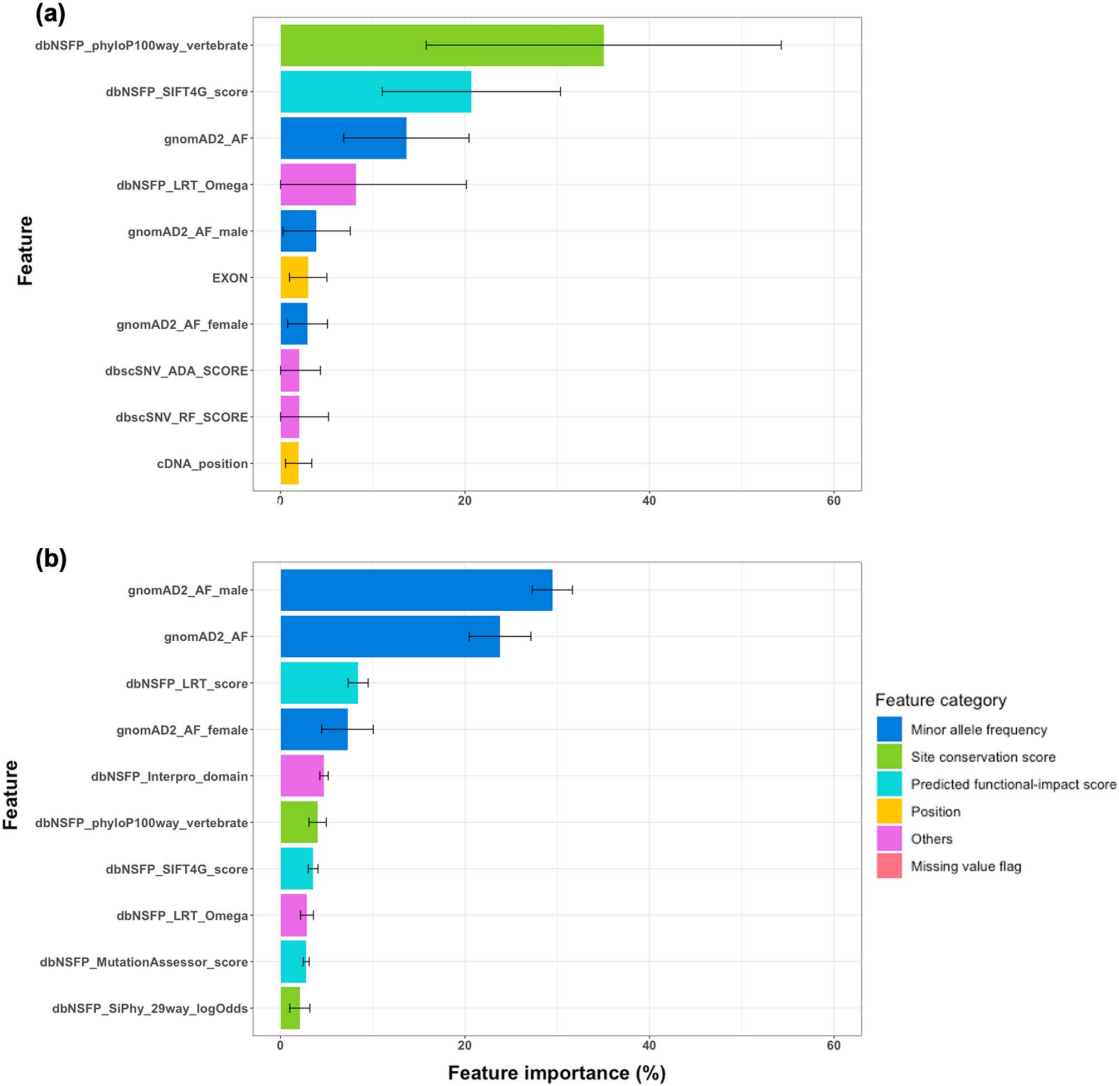
Top ten important features of extreme gradient boosting models for *BRCA1* trained using (**a**) gene-specific and (**b**) disease-specific variants. The categories of features are shown using different colours. Feature importance values averaged over the ten trials (see Methods) are shown with error bars.

Figure 4 shows the top ten important features identified by gene-specific and disease-specific learning of XGBoost for *BRCA1*. The XGBoost feature importance values of all features in the ten trials are shown in Supplementary Tables S9 and S10, respectively, for gene-specific and disease-specific learning. In the gene-specific and disease-specific XGBoost models for *BRCA1*, the top ten important features had 93.3% and 88.9% of the feature importance values, respectively. In addition, we observed that variance across the ten trials was much higher for gene-specific learning than disease-specific learning, possibly due to the smaller size of the gene-specific training dataset (see Methods). The most important feature learned from *BRCA1*-specific training variants was dbNSFP_phyloP100way_vertebrate (a site conservation score; feature importance 35.03 ± 19.24%). The second and third were dbNSFP_SIFT4G_score (a predicted functional-impact score; feature importance 20.69 ± 9.66%) and gnomAD2_AF (a MAF; feature importance 13.64 ± 6.80%). Compared to this, the most critical feature learned from disease-specific variants was gnomAD2_AF_male (a MAF; feature importance 29.46 ± 2.18%). The second was gnomAD2_AF (a MAF; feature importance 23.80 ± 3.34%). The third was dbNSFP_LRT_score (a predicted functional-impact score; feature importance 8.41 ± 1.09%). The two most important features learned by gene-specific learning for *BRCA1* were site conservation and predicted functional-impact scores. In contrast, the first and second important features in the disease-specific XGBoost models for *BRCA1* were MAF features, i.e., gnomAD2_AF_male and gnomAD2_AF.

We observed similar trends when comparing the top ten important feature groups. The top ten important feature groups of the *BRCA1*-specific and disease-specific XGBoost models shared six features. Differences between the two important feature groups were as follows (see Fig. 4). Ranks of MAF features were higher (first, second, and fourth) in disease-specific learning compared to *BRCA1*-specific learning (third, fifth, and seventh). It seems that *BRCA1*-specific training variants were insufficient to learn a reliable pattern of MAFs for discriminating between pathogenic and benign variants compared to disease-specific training variants. Two genomic position features, i.e., EXON and cDNA_position, were among the top ten crucial features in gene-specific learning. However, the feature importance values of these features in disease-specific learning for *BRCA1* were much lower (ranked 27th and 24th, respectively; see Supplementary Table S10). The position features exhibit relatively high importance values in gene-specific learning, likely due to the fact that positional information is only meaningful within a specific gene and not applicable across a group of genes, even if they are linked to the same or similar diseases.

**Figure 5:**
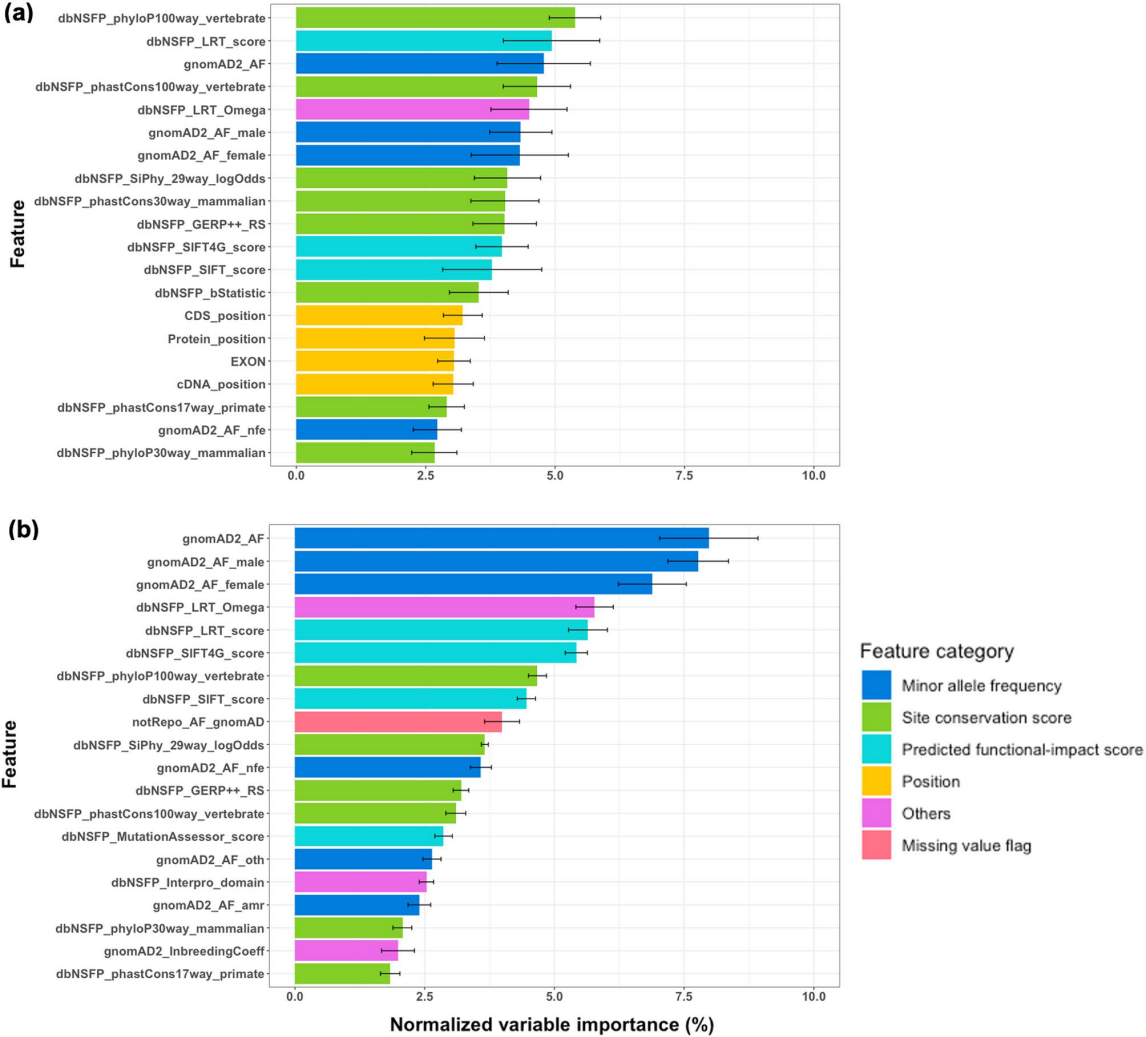
Top twenty important features of random forest models for *BRCA2* trained using (**a**) gene-specific and (**b**) disease-specific variants. The categories of features are shown using different colours. Normalized variable importance values averaged over the ten trials (see Methods) are shown with error bars.

Figure 5 shows the most critical twenty features learned from gene-specific and disease-specific RF learning for *BRCA2*. We offer all features’ normalized variable importance values in Supplementary Tables S11 and S12 for *BRCA2*-specific and disease-specific learning, respectively. Variable importance values of RF were normalized so that their sum over all features equals 100%. The top twenty features had 76.9% and 82.4% of the variable importance values for gene-specific and disease-specific RF learning for *BRCA2*, respectively. Similar to the result for *BRCA1*, we observed that variance across the ten trials was generally higher for gene-specific RF learning than disease-specific RF learning, possibly due to the smaller training dataset size of gene-specific learning. The three most important features for disease-specific RF learning for *BRCA2* were gnomAD2_AF (a MAF; normalized variable importance 7.98 ± 0.95%), gnomAD2_AF_male (a MAF; normalized variable importance 7.77 ± 0.58%), and gnomAD2_AF_female (a MAF; normalized variable importance 6.89 ± 0.66%). Among these three MAF features, only gnomAD2_AF was among the top three critical features for *BRCA2*-specific RF learning (ranked third; normalized variable importance 4.78 ± 0.90%). The first and second essential features for *BRCA2*-specific RF learning were dbNSFP_phyloP100way_vertebrate (a site conservation score; normalized variable importance 5.38 ± 0.50%) and dbNSFP_LRT_score (a predicted functional-impact score; normalized variable importance 4.93 ± 0.93%), respectively. We note that dbNSFP_phyloP100way_vertebrate was also the most crucial feature learned from *BRCA1*-specific XGBoost training (see Fig. 4a). It suggests the site conservation score is a critical gene-specific information source for discriminating between pathogenic and benign variants.

The comparison results of the top twenty feature groups between *BRCA2*-specific and disease-specific RF learning are as follows. Fourteen features were common among the top twenty gene-specific and disease-specific feature groups obtained from RF learning for *BRCA2*. However, each feature’s rank differed, meaning that different optimal RF models were learned from *BRCA2*-specific and disease-specific variants, respectively. We observed that MAF features were more influential in disease-specific learning (ranked first (gnomAD2_AF), second (gnomAD2_AF_male), third (gnomAD2_AF_female), 11th (gnomAD2_AF_nfe), 15th (gnomAD2_AF_oth), and 17th (gnomAD2_AF_amr)) than in *BRCA2*-specific learning. Ranks of the same MAF features were lower in *BRCA2*-specific RF learning: 3rd, 6th, 7th, 19th, 22nd, and 29th, respectively (see Supplementary Table S11). This result is similar to that from XGBoost learning for *BRCA1* (see Fig. 4). Another similar result is that position features were more critical in *BRCA2*-specific learning than disease-specific learning. In *BRCA2*-specific RF models, ranks of the four position features, i.e., EXON, cDNA_position, CDS_position, and protein_position, were 16th, 17th, 14th, and 15th, respectively. On the contrary, the same features were ranked 26th, 27th, 29th, and 28th in disease-specific RF models for *BRCA2* (see Supplementary Table S12).

**Figure 6:**
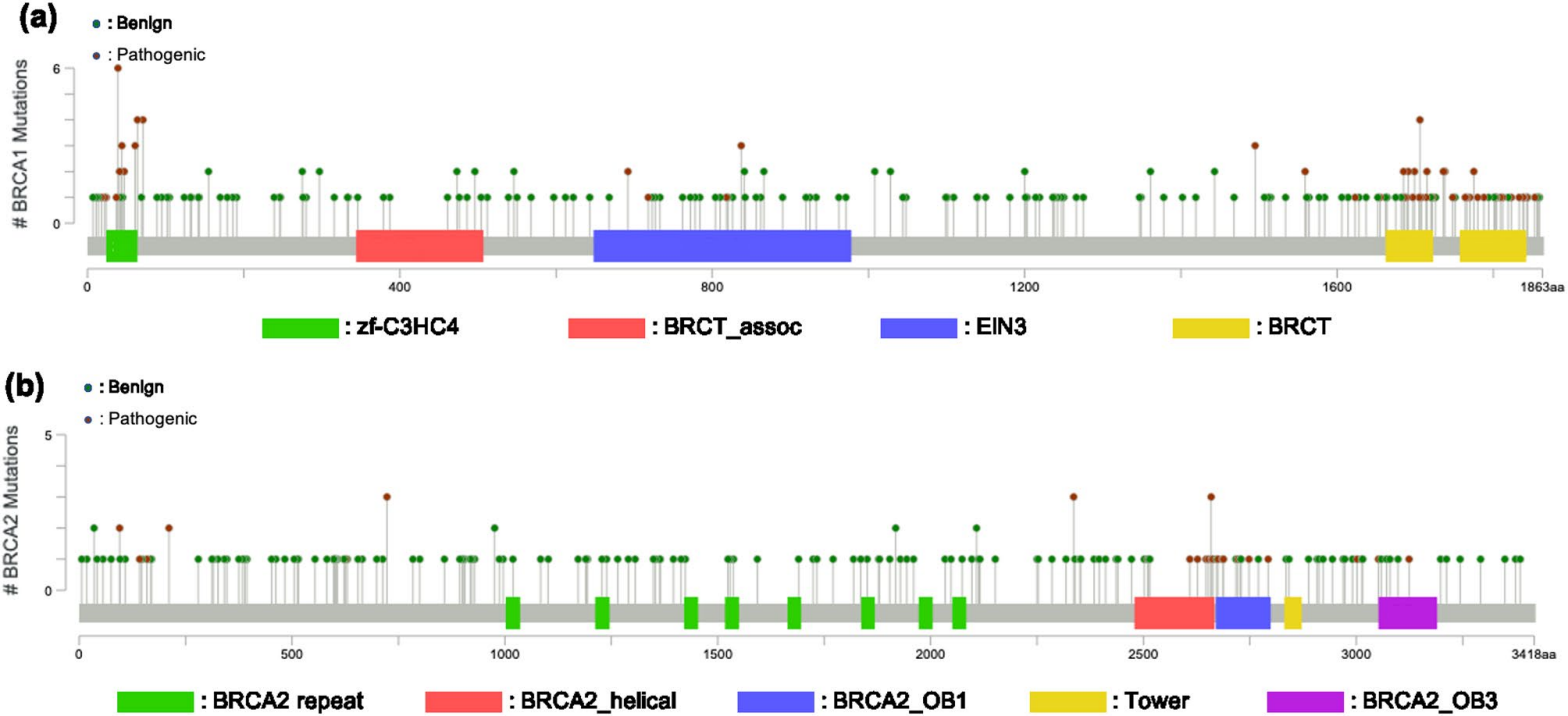
Location of (**a**) *BRCA1* and (**b**) *BRCA2* variants in the amino acid sequences. Pathogenic (including Pathogenic, Pathogenic/Likely_pathogenic, and Likely_pathogenic) and benign (including Benign, Benign/Likely_benign, and Likely_benign) variants are shown in different colours.

To summarize, we observed common properties in important features identified by gene-specific and disease-specific learning for the pathogenicity prediction of rare *BRCA1/2* missense variants. First, MAF features were more critical in disease-specific learning than gene-specific learning. It means that MAF is a major discriminating factor between pathogenic and benign variants, having similar patterns regardless of genes, at least if they are associated with the same disease. However, gene-specific training variants seem insufficient to capture the discriminating pattern reliably. Instead of MAF features, we can use predicted functional-impact and site conservation scores as significant elements for distinguishing between pathogenic and benign variants, as shown by the optimal performance of gene-specific learning. Additionally, the position of a variant could play an essential role only in gene-specific learning because the meaning of position could be different by genes. Figure 6 shows the position of each variant of *BRCA1/2* used in our experiments. It can be seen that the region enriched for pathogenic variants differs between the two genes.

## Conclusions

Machine learning has shown promise in tackling the challenge of interpreting rare missense variants in disease-associated genes, such as *BRCA1* and *BRCA2*. Choosing the appropriate set of training variants is crucial for developing an accurate pathogenicity predictor using machine learning. Studies have found that gene-specific and disease-specific approaches are more effective than genome-wide approaches. We conducted a study comparing gene-specific and disease-specific machine learning methods for predicting the pathogenicity of rare missense variants in *BRCA1/2*. Our findings suggest that gene-specific machine learning can achieve optimal pathogenicity prediction with an appropriate algorithm, without the need to include disease-specific variants in the training set.

We acknowledge that aspects other than the composition of the training variant datasets, such as feature selection and data balancing, have an impact on the efficiency and effectiveness of pathogenicity prediction. In particular, data balancing could be a good option considering the fact that the pathogenic and benign variant datasets are usually imbalanced. In the present work, we did not apply the data balancing technique because there is a controversy about its effectiveness. For example, Kim and Hwang have shown that most over- and undersampling methods for data balancing were ineffective or even reduced the performance of a classifier^50^. Of course, it would be a promising further research direction to investigate the effect of data balancing methods on pathogenicity prediction of rare *BRCA1/2* missense variants. Another direction of research is to include more gene-level features, such as mutational signatures and biological pathway/signaling network information. There has been a study demonstrating the effectiveness of expression quantitative trait loci in predicting the disease relevance of non-coding variants^51^. These features could also improve the performance of pathogenicity prediction of rare *BRCA1/2* missense variants.

Some machine learning algorithms produced the best predictor regardless of the type of training variant set. MAF features were more important in disease-specific predictors, while position features played a significant role in gene-specific predictors. These results indicate that gene-specific machine learning, utilizing gene-specific variant characteristics, can produce the optimal pathogenicity predictor for *BRCA1* and *BRCA2*, despite the limited size of the training dataset. Therefore, we recommend using gene-specific machine learning over disease-specific learning for predicting the pathogenicity of rare missense variants in *BRCA1/2* because it is efficient and effective, with the caveat that gene-specific approaches may not be applicable for genes with extremely low numbers of variants, in which case disease-specific approaches may be more appropriate.

## Supplementary Information

Supplementary Table S1.

Supplementary Table S2.

Supplementary Table S3.

Supplementary Table S4.

Supplementary Table S5.

Supplementary Table S6.

Supplementary Table S7.

Supplementary Table S8.

Supplementary Table S9.

Supplementary Table S10.

Supplementary Table S11.

Supplementary Table S12.

## Data Availability

The ClinVar variant file (clinvar_20200817.vcf.gz) was downloaded from https://ftp.ncbi.nlm.nih.gov/pub/clinvar/vcf_GRCh37/archive_2.0/2020/. The dbSNP (build 151) variant file (All_20180423.vcf.gz) was downloaded from https://ftp.ncbi.nih.gov/snp/organisms/human_9606_b151_GRCh37p13/VCF/. The dbscSNV (version 1.1) variant file (dbscSNV1.1.zip) was downloaded from http://www.liulab.science/dbscsnv.html. The gnomAD (release 2.1.1) variant file (gnomad.exomes.r2.1.1.sites.vcf.bgz) was downloaded from https://storage.googleapis.com/gcp-public-data--gnomad/release/2.1.1/vcf/exomes/gnomad.exomes.r2.1.1.sites.vcf.bgz. The KOVA variant file (K1055E_allele_frequency.txt.zip) was downloaded from http://kobic.re.kr/kova/downloads. The KRGDB (phase 2) variant files (KRG1100_rare_variants.zip and KRG1100_common_variants.zip) were downloaded from http://coda.nih.go.kr/coda/KRGDB/index.jsp. The dbNSFP (version 4.1a) data file (dbNSFP4.1a.zip) was downloaded from https://drive.google.com/file/d/17kdX1Fqi_ZW8PXaHm2vQuJLHuoMDwZmB/view.
